# Visuo-Cognitive Phenotypes in Early Multiple Sclerosis: A Multisystem Model of Visual Processing

**DOI:** 10.3390/jcm13030649

**Published:** 2024-01-23

**Authors:** Hariklia Vagias, Michelle L. Byrne, Lyn Millist, Owen White, Meaghan Clough, Joanne Fielding

**Affiliations:** 1 School of Psychological Sciences and the Turner Institute for Brain and Mental Health, Monash University, Melbourne 3800, Australia; hariklia.vagias1@monash.edu (H.V.);; 2 Department of Neuroscience, Alfred Hospital, Melbourne 3004, Australia; 3 Department of Neuroscience, Central Clinical School, Monash University, Melbourne 3004, Australiajoanne.fielding@monash.edu (J.F.)

**Keywords:** multiple sclerosis, phenotypes, cognition, visual processing, visuo-cognitive

## Abstract

Background: Cognitive impairment can emerge in the earliest stages of multiple sclerosis (MS), with heterogeneity in cognitive deficits often hindering symptom identification and management. Sensory–motor dysfunction, such as visual processing impairment, is also common in early disease and can impact neuropsychological task performance in MS. However, cognitive phenotype research in MS does not currently consider the relationship between early cognitive changes and visual processing impairment. Objectives: This study explored the relationship between cognition and visual processing in early MS by adopting a three-system model of afferent sensory, central cognitive and efferent ocular motor visual processing to identify distinct visuo-cognitive phenotypes. Methods: Patients with clinically isolated syndrome and relapsing–remitting MS underwent neuro-ophthalmic, ocular motor and neuropsychological evaluation to assess each visual processing system. The factor structure of ocular motor variables was examined using exploratory factor analysis, and phenotypes were identified using latent profile analysis. Results: Analyses revealed three ocular-motor constructs (cognitive control, cognitive processing speed and basic visual processing) and four visuo-cognitive phenotypes (early visual changes, efferent-cognitive, cognitive control and afferent-processing speed). While the efferent-cognitive phenotype was present in significantly older patients than was the early visual changes phenotype, there were no other demographic differences between phenotypes. The efferent-cognitive and cognitive control phenotypes had poorer performance on the Symbol Digit Modalities Test compared to that of other phenotypes; however, no other differences in performance were detected. Conclusion: Our findings suggest that distinct visual processing deficits in early MS may differentially impact cognition, which is not captured using standard neuropsychological evaluation. Further research may facilitate improved symptom identification and intervention in early disease.

## 1. Introduction

Cognitive impairment is a prevalent symptom of multiple sclerosis (MS) that can profoundly impact quality of life and lead to participation restriction [1,2]. Often arising early in the disease, including clinically isolated syndrome, initial cognitive symptoms may precede detectable structural changes on neuroimaging and are associated with poorer clinical outcomes [3,4]. While processing speed, working memory and executive functioning are commonly affected cognitive domains, there is significant heterogeneity in MS-related cognitive deficits, which can impede symptom identification and management [5,6]. Recent studies have explored cognitive phenotypes in more advanced stages of MS to identify homogenous subgroups of patients with shared cognitive impairments [7,8,9,10]. While this phenotype approach may offer utility in the development of individually tailored treatment options, it does not currently consider the relationship between early cognitive impairment and deficits in other physiological systems, such as sensory and motor function. Notably, visual processing, our most dominant sense, can impact performance on current gold-standard measures of cognition in MS [11,12]. Consequently, adopting a multisystem approach to identify phenotypes that capture both visual processing and cognitive function in early MS may help to facilitate earlier symptom identification and improve the efficacy of personalised intervention.

At least one-third of people with MS experience persistent visual deficits [13], which are recognised as the second largest contributor to reduced quality of life [14]. Much like cognitive symptoms, visual deficits are typically one of the first symptoms to appear [15], including thinning of the retinal nerve fibre layer (RNFL), which is associated with disease and disability progression [16]. Current research in MS has focused primarily on deficits related to the transmission of visual information from the retina to the visual cortex (i.e., the sensory afferent system), such as optic neuritis, and the subsequent generation of an eye movement to redirect the retinal fovea (i.e., the efferent ocular motor system), such as internuclear ophthalmoplegia (INO). However, the processing of visual information implicates a far wider visual processing network that extends to the cognitive integration of sensory information to perceive a meaningful image and the incorporation of this information with personal goals and intentions to inform a behaviourally relevant response (i.e., the central cognitive system). Thus, visual processing does not merely involve the ability of the eyes to detect visual stimuli; it encompasses the expansive neurological processes that shape our perception of the world around us and enable meaningful interaction with our environment, such as through recognising faces, reading, writing and safely navigating our surroundings. In MS, cognitive visual processing deficits related to processing speed [17], spatial working memory [18] and executive control [19] can impede an individual’s ability to effectively engage with their environment in everyday life.

An inextricable link exists between visual processing and cognition. In the context of MS, there is a known association between RNFL thinning and cognitive impairment [20], as well as evidence to suggest that decreased connectivity between occipital regions is associated with reduced cognitive function [21]. While these findings indicate a complex relationship between the visual network and cognitive impairment in MS, the nature of this relationship is poorly understood [22]. Specifically, little is known about how deficits related to central cognitive visual processing impact the cognitive functions that intricately rely upon this system and how this relates to afferent and efferent visual processing. Research is therefore needed to delineate the independent and combined influence of these visual processing systems on cognition in MS. However, it is difficult to determine the independent effects of visual and cognitive deficits using standard neuropsychological assessments, such as the Symbol Digit Modalities Test (SDMT), which can be influenced by visual and motor impairment in MS [11,12,23]. For example, an MS patient with residual visual acuity deficits after an acute episode of optic neuritis may have difficulty with distinguishing symbols on the SDMT, while a patient with INO may experience double or blurred vision when visually scanning the reference key at the top of the page. Auditory-based neuropsychological tasks have been proposed as alternatives to avoid the confounders of visual and motor impairment upon cognitive assessment; however, these measures are susceptible to oral motor slowing in MS [24]. Contrastingly, ocular motor (OM) assessment can be used to dissociate cognitive visual processing from sensory–motor visual processing in MS [17].

To address the limitations of previous research, this exploratory study proposes a novel three-system model of afferent sensory, central cognitive and efferent ocular motor visual processing to examine the relationship between cognitive and visual processing deficits in the early stages of MS. This multi-system conceptualisation offers an opportunity to dissociate each visual processing system and explore both their independent and joint impact on cognition. Using OM assessment, in conjunction with clinical measures of afferent and efferent visual processing, we sought to identify distinct visuo-cognitive phenotypes in MS and examine their clinical significance.

## 2. Materials and Methods

### 2.1. Participants

This study included 165 participants, 50 patients with clinically isolated syndrome (CIS), 90 patients with clinically definite MS (CDMS) of a relapsing remitting course and 25 healthy control participants who were recruited from the community. All CIS patients had experienced an initial neurological event and had MRI pathology consistent with demyelination. All CDMS patients had received a diagnosis of MS in accordance with the McDonald diagnostic criteria [25]. Exclusion criteria for patients included experiencing a clinical relapse at the time of testing or being treated with steroids within the previous three months. Over the course of the study, approximately 84% of CDMS patients were taking disease-modifying treatments (61% Fingolimod, 15% Interferon Beta-1a, 5% Interferon Beta-1b, 5% Glatiramer Acetate, 5% Cladribine, 3.5% Peginterferon Beta-1a, 3.5% Dimethyl Fumarate and 2% Natalizumab) while 16% of patients were untreated. The majority of CIS patients were untreated over the course of the study, with only 44% of patients taking disease-modifying treatments (50% Fingolimod, 25% Interferon Beta-1a, 10% Glatiramer Acetate, 5% Interferon Beta-1b, 5% Peginterferon Beta-1a and 5% Natalizumab). Exclusion criteria for controls included a history of head injury, a neurological condition, a psychiatric disorder, substance use disorder or regular psychoactive drug use.

### 2.2. Study Design

This exploratory study adopted a retrospective cross-sectional design. It comprises clinical patient data collected during routine neuro-ophthalmic assessment and research data (including ocular motor and neuropsychological assessment) collected as part of previous studies [18,26]. Patients attended both a clinical and research visit, while control participants only completed a research visit. Please see Table 1 for further information about measures completed during these visits.

### 2.3. Afferent Visual Processing Measures

#### 2.3.1. Retinal Nerve Fibre Layer Thickness (RNFL)

Optical coherence tomography (OCT) was conducted and analysed by trained neurophysiologists using a Heidelberg Engineering Spectralis OCT device. The key outcome measure was global RNFL thickness (μm: internal limiting membrane to RNFL/ganglion cell layer), and the eye with a thinner global RNFL value was used in analyses.

#### 2.3.2. Visual Acuity

Visual acuity was assessed by a neuro-ophthalmologist using the Snellen chart. Visual acuity scores were converted to a log of minimum angle of resolution (LogMAR) format for statistical analysis, per Tiew et al. [27]. To ensure consistency across afferent measures, the eye chosen for RNFL measurement was selected for visual acuity. For patients lacking RNFL data, the eye with worse visual acuity was selected for analyses.

### 2.4. Cognitive Visual Processing Measures

#### 2.4.1. Ocular Motor Apparatus

The Eyelink II dark pupil video-oculography system (SR-Research Ltd., Mississauga, ON, Canada), which has a high resolution and high acquisition rate (500 Hz), was used to record horizontal displacement of the eye under different experimental conditions. Screen-based stimuli were generated using Experiment Builder (version 1.10.165) and displayed on a 22-inch CRT monitor, as previously described by Clough et al. [18]. Participants were seated 840 mm in front of the monitor in a darkened room.

#### 2.4.2. Ocular Motor Tasks

**Figure 1:**
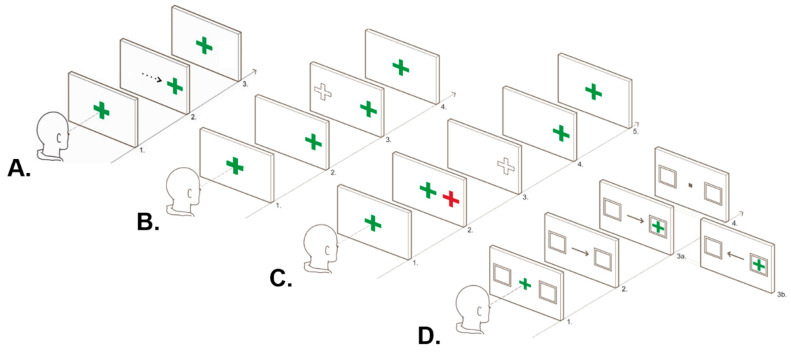
**Diagram of ocular motor tasks.** (**A**). Visually guided task: 1. Participants initially fixated on a central green cross. 2. A saccade was then performed to a randomly appearing visual stimulus, which shifted from the centre to 5 or 10 degrees left and right of the centre over 24 trials. 3. Gaze was reoriented to the centre with the presentation of the central green cross. (**B**). Antisaccade task: 1. Participants initially fixated on a central green cross. 2. The central green cross disappeared concurrently with the appearance of a green cross either 5 or 10 degrees to the left or right of the centre over 48 trials. 3. Participants were instructed to inhibit a reflexive saccade to the suddenly appearing peripheral green cross and instead generate an equal-amplitude saccade in the mirror opposite direction (the correct response is depicted by a dotted-line cross). 4. Gaze was reoriented back to the centre when the central green cross re-appeared. (**C**). Memory-guided task: 1. Participants initially fixated on a central green cross. 2. A red cross appeared for 500 ms and participants were instructed to remember the spatial location of the red cross without looking at it. 3. Following 1500 or 2500 ms, the central cross disappeared and participants were required to generate a saccade to the approximate spatial location of the previously presented red cross (the correct response is depicted by a dotted-line cross). 4. A green cross was then presented in the same location as the red target cross to allow participants to adjust their final eye position. 5. Gaze was reoriented to the centre with the presentation of a central green cross. (**D**). Endogenously cued task: 1. Participants were required to fixate on a central green cross. 2. After 850 ms, this cross was replaced with an arrow pointing to a peripheral box on either the righthand or lefthand side for 500 ms. 3. Participants were instructed to shift their gaze towards a green cross when it appeared in one of the two boxes, with the arrow accurately predicting the location of the peripheral target in 75% of trials (48 trials in total). Valid trials are denoted by the arrow accurately predicting the location of the peripheral stimulus (3a), while invalid trials are denoted by the arrow pointing in the opposite direction (3b). 4. Gaze was reoriented to the centre with the presentation of a central black box. Adapted from Clough [28].

The visually guided (VG) task, a basic prosaccade task, was administered to measure simple visual processing due to its minimal cognitive load. Three cognitive ocular motor tasks were also administered. This included the anti-saccade (AS) task, a measure of inhibitory control and spatial working memory; the memory guided (MG) task, a measure of spatial working memory and inhibitory control; and the endogenously cued (EC) task, a measure of attentional control. Full task details are summarised in Figure 1.

#### 2.4.3. Ocular Motor Data Processing

Monocular saccade analysis was conducted using the right eye for each participant, except for instances of INO or observable dysconjugacy where the unaffected eye was analysed. A semi-automated program written in Matlab was used to extract specific saccadic metrics (see Supplemental Materials, Section S1.1, for further details). Key ocular motor measures included latency, proportion of errors and final eye position.

Saccadic latency represents the temporal difference between stimulus onset and saccade onset for correct trials only. Per Clough et al. [17], latencies from tasks with a higher cognitive load (i.e., EC, AS and MG tasks) were adjusted by VG task latency using linear regression to dissociate complex processing speed from simple processing speed on each task. These adjusted latencies are referred to as EC latency, AS latency and MG latency herein.

Task errors (EC error, AS error and MG error) were calculated as a percentage of total trials and were determined according to the task-specific rules described in the Supplementary Materials (Section S1.2). Final eye position (FEP) is a measure of spatial accuracy and was calculated for the AS and MG tasks (AS FEP and MG FEP).

### 2.5. Efferent Visual Processing Measures

#### 2.5.1. Diagnosis of Eye Movement Disorder

The clinical evaluation of eye movement abnormalities was conducted by a neuro-ophthalmologist during routine ophthalmic assessment. Nine patients in the current study were found to have clinical INO, 5 with unilateral INO and 4 with bilateral INO. No patient experienced nystagmus or oscillopsia at the time of the assessment.

#### 2.5.2. Versional Dysconjugacy Index

Subclinical INO was assessed using binocular saccade data from the VG task to calculate the versional dysconjugacy index (VDI), per the procedure established by Bijvank et al. [29]. This involved the derivation of a dysconjugacy ratio of the abducting and adducting eye for both rightward (VDI_R) and leftward (VDI_L) saccades (see Section S1.3 of the Supplementary Materials for further details). The VDI was calculated for all patients irrespective of clinical INO diagnosis.

### 2.6. Clinical Characteristics

#### 2.6.1. Neuropsychological Assessment

The oral SDMT, National Adult Reading Test (NART) and Beck Depression Inventory (BDI) were administered during each research visit as part of a wider battery of neuropsychological tasks. The oral SDMT measures an array of cognitive functions, including visual attention and processing speed [30], and was used in this study as a screening measure of cognitive dysfunction. Higher scores on the SDMT reflect superior performance. The NART was used as a measure of premorbid intellectual functioning [31], with scores representing an age-normalised standard score. The BDI was used as a self-report measure of current depressive symptoms, with higher scores indicative of more severe depressive symptomology [32].

#### 2.6.2. Assessment of Disease and Disability Severity

The Expanded Disability Status Scale (EDSS) was administered by a neuro-ophthalmologist during each patient’s clinical visit and was used as a measure of disability severity, with a higher total score indicating a greater disability burden. During this visit, information regarding disease status and the patient’s subjective account of the first symptom’s onset was also recorded.

### 2.7. Data Preparation

Most visual processing variables were found to have non-normal distributions (see Supplementary Materials, Section S2.1, for further details). Non-parametric tests were used as alternatives to parametric analyses when the assumption of normality was required, and robust model estimators were employed. Patient OM data were z-scored against the mean and standard deviation of healthy control participants to allow for interpretation on a consistent scale and to quantify the severity of impairment in patients relative to healthy individuals. As OCT was not conducted in the control group, the RNFL was z-scored using normative data [33].

There were missing data for some visual processing measures, particularly the MG task (full details can be found in Section S2.2 of the Supplementary Materials). Among individuals with missing data for the MG task, there were some significant differences in other variables compared to those without missing data. However, much of this missingness was considered to be random (MAR) due to a temporary change in protocol where the MG task was not administered. To retain power and avoid bias, multiple imputation was used to generate plausible values for missing data. Please refer to Section S2.3 of the Supplementary Materials for a description of this process.

### 2.8. Data Analysis

Statistical analysis was conducted in Mplus version 8.9 [34], R version 4.2.2 [35] and RStudio version 2022.07.2 [36]. The specific code used for analyses can be found via the following link: https://doi.org/10.5281/zenodo.10086915 (created on 9 November 2023).

#### 2.8.1. Exploratory Factor Analysis

Exploratory factor analysis (EFA) was conducted using the R package lavaan (version 0.6-12) [37] to examine the underlying factor structure of ocular motor variables. Models with 1–6 factors were run using maximum likelihood (ML) estimation with oblique rotation. These models were evaluated by comparing various fit indices including the chi-square test, comparative fit index (CFI), Tucker–Lewis index (TLI), root mean square error of approximation (RMSEA) and standardised root mean square residual (SRMR). Given the non-normal distribution of most variables, robust estimates were used for each fit index. Indicators with significant factor loadings above 0.4 were then examined within each model. While model selection was guided by a comparison of fit indices, the interpretability of factors and theoretical considerations were also taken into account (see Supplementary Materials, Section S3, for further details).

#### 2.8.2. Latent Profile Analysis

Latent profile analysis (LPA) was used as a data-driven approach to identify visuo-cognitive phenotypes in MS based on deficits across the systems of afferent, cognitive and efferent visual processing. LPA was conducted in Mplus and involved a multi-step process, as follows:

1. Selection of variables. The variables of RNFL, VG latency, EC latency, EC error, AS latency, AS FEP, AS error, MG latency, MG FEP, MG error, VDI_R and VDI_L were included in the LPA models. Visual acuity was initially included in models following LogMAR conversion. However, the inclusion of visual acuity negatively impacted the sample size and interpretability of generated phenotypes, despite the comparable visual acuity estimates between profiles. Consequently, visual acuity was removed from the LPA model and treated as a demographic variable for post-hoc analyses.
2. Model specification and selection. Given the non-normal distribution of most visual processing variables, maximum likelihood with robust standard errors (MLR) was applied to latent profile models [38]. There was no a priori assumption regarding how many phenotypes exist due to the lack of previous research on visuo-cognitive phenotypes in MS. Therefore, models with 1 to 6 profiles were sequentially run and model fit indices were examined to determine the best fitting model, including the Bayesian information criterion and the bootstrap likelihood ratio test [39]. The theoretical and clinical interpretability of each model was also considered in the selection process. Please see Supplementary Materials, Section S4.1, for further details regarding model selection. To avoid local maxima, 250 sets of starting values were used for the initial maximisation stage and the 50 best solutions were retained for final-stage optimisation in each model [38]. The highest log-likelihood value was successfully replicated in our selected model, indicating that a global solution was found.
3. Model interpretation. Results of the EFA were used to inform our interpretation of phenotypes, with qualitative descriptors assigned to profiles based on the unique visual processing deficits that differentiated each phenotype. As patient data were standardised against those of healthy controls, a value of zero represented the healthy control mean. Thus, z-scores between 1 and 2 represented a mild impairment, z-scores between 2 and 3 represented a moderate impairment and z-scores greater than 3 represented a severe impairment for OM variables. RNFL was inversely interpreted, with z-scores below −1 denoting afferent visual processing impairment.
4. Sensitivity analysis. Given the asymmetrical distribution of imputed values for some missing datapoints, a sensitivity analysis was conducted by running another series of LPA models with pairwise missingness to compare against imputed LPA models. Please see Section S4.2 of the Supplementary Materials for further details.
5. Phenotype differences. For the selected LPA model, the entropy was 0.94 and all average latent class probabilities were greater than 0.9, suggesting that the generated profiles could be treated as discrete categories for post hoc analyses [39]. Thus, inter-phenotype differences across various clinical characteristics were examined using a Kruskal–Wallis test with Dunn’s multiple comparison to gain further insight into the clinical relevance of each phenotype.

## 3. Results

### 3.1. Descriptive Statistics

As outlined in Table 2, CDMS patients were found to be significantly older than both healthy controls and CIS patients (omnibus F (2) = 11.01, *p* < .001, *η*^2^ = 0.12). While healthy controls had a higher estimated premorbid IQ than did CIS patients (omnibus F (2) = 5.72, *p* = .004, *η*^2^ = 0.07), the mean premorbid IQ was in the high average range across all three groups. Healthy controls had significantly lower self-reported depressive symptoms compared to patient groups (omnibus F (2) = 4.12, *p* = .02, *η*^2^ = 0.05); however, average self-reported depressive symptoms were still within normal limits for CIS and CDMS groups. There were no significant differences in SDMT performance between control and patient groups. CDMS patients were found to have a longer symptom duration (W = 384.5, *p* < .001, *d* = −0.62) and higher EDSS score (W = 1249.5, *p* = .006, *d* = −0.24) than those of CIS patients, while visual acuity scores did not differ between patient groups.

In the overall patient sample, most visual processing variables had weak or non-significant correlations with demographic characteristics. However, AS error was positively associated with age (*r_s_* = 0.37, *p* < .001) and EDSS score (*r_s_* = 0.33, *p* < .001), while RNFL was negatively correlated with EDSS score (*r_s_* = −0.37, *p* < .001) and symptom duration (*r_s_* = −0.41, *p* < .001).

Healthy controls had significantly shorter latency on the VG task (W = 1037, *p* = .001, *d* = −0.41) and a lower error rate across the EC (W = 991, *p* < .001, *d* = −0.43), AS (W = 1119, *p* = .005, *d* = −0.35) and MG (W = 890.5, *p* = .009, *d* = −0.34) tasks compared with those of the overall patient group. Controls and patients did not significantly differ with regard to VDI or latency and final eye position across cognitive OM tasks, although the patient group consistently demonstrated a wider range of values, with notably higher upper limits than those of the control group (see Table 3).

In the patient group, AS latency was positively correlated with EC latency (*r_s_* = 0.30, *p* < .001) and MG latency (*r_s_* = 0.31, *p* < .001). EC latency and MG latency also had a significant positive association (*r_s_* = 0.36, *p* < .001). AS error had a significant positive association with EC error (*r_s_* = 0.34, *p* < .001) and MG error (*r_s_* = 0.54, *p* < .001), as did EC error and MG error (*r_s_* = 0.36, *p* < .001). All other indicators either had a non-significant or weak correlation with one another.

### 3.2. Exploratory Factor Analysis

**Figure 2:**
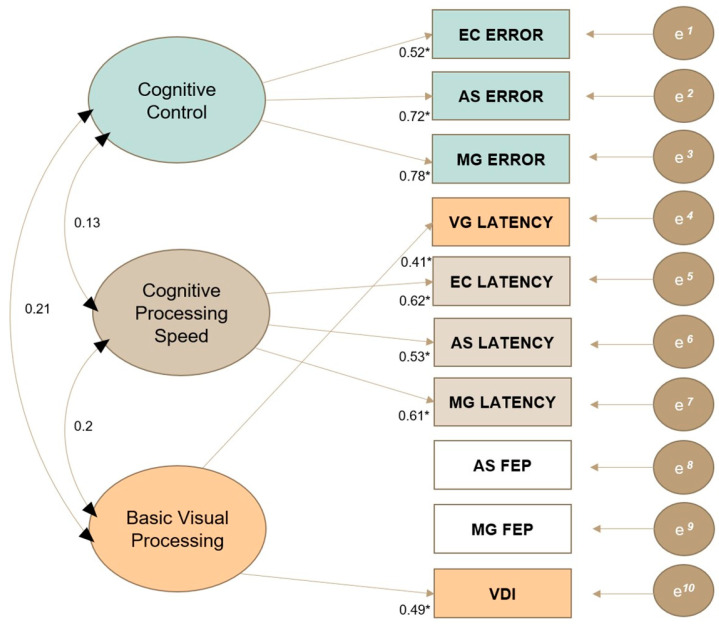
**Exploratory factor analysis: 3-factor model of ocular motor variables.** Abbreviations: VG, visually guided; EC, endogenously cued; AS, antisaccade; MG, memory guided; FEP, final eye position; VDI, versional dysconjugacy index, e*^1–10^*; measurement error for each of the 10 indicators. * *p* < .05.

A three-factor model provided the most interpretable solution for the underlying structure of ocular motor variables, with robust fit indices of *χ*^2^ = 23.64, *df* = 18, *p* = .17, CFI = 0.96, TLI = 0.9, RMSEA = 0.05 and SRMR = 0.03. As outlined in Figure 2, the following qualitative labels were assigned to each factor based on the underlying construct shared among indicators that significantly loaded onto that factor: (1) cognitive control, (2) cognitive processing speed and (3) basic visual processing. These factors were used to inform the interpretation of phenotypes generated via LPA. AS FEP and MG FEP did not significantly load onto any factor in the EFA. However, these variables were retained in the final model as their exclusion led to worse fit indices and factor loadings of other indicators, suggesting that they contribute to the overall measurement of latent OM constructs despite their non-significant loadings.

### 3.3. Latent Profile Analysis

A four-profile model provided the best fit to the data, with profile estimates summarised in Table 4. The first profile, termed the ‘Early Visual Changes’ phenotype (*n* = 100, 82% female, 56% CDMS, mean age = 38.72), only subtly deviated from healthy controls across each visual processing estimate. The second profile, termed the ‘Efferent-Cognitive’ phenotype (*n* = 12, 83% female, 92% CDMS, mean age = 47.78), had mild to severe cognitive visual processing deficits across the domains of basic processing speed, cognitive control and spatial accuracy, as well as severe unilateral dysconjugacy, compared with healthy controls. Notably, six of the nine individuals with clinically diagnosed INO in the overall sample were included in this phenotype. The third profile, termed the ‘Cognitive Control’ phenotype (*n* = 12, 83% female, 83% CDMS, mean age = 46.73), was characterised by moderate to severe cognitive control deficits across the three cognitive OM tasks. This phenotype did not substantially differ from healthy controls regarding afferent and efferent visual processing. The final profile, termed the ‘Afferent-Processing Speed’ phenotype (*n* = 16, 88% female, 81% CDMS, mean age = 41.48), was characterised by a mild structural afferent deficit and mild to moderate deficits in both basic and cognitive processing speed, compared to that of healthy controls.

**Figure 3:**
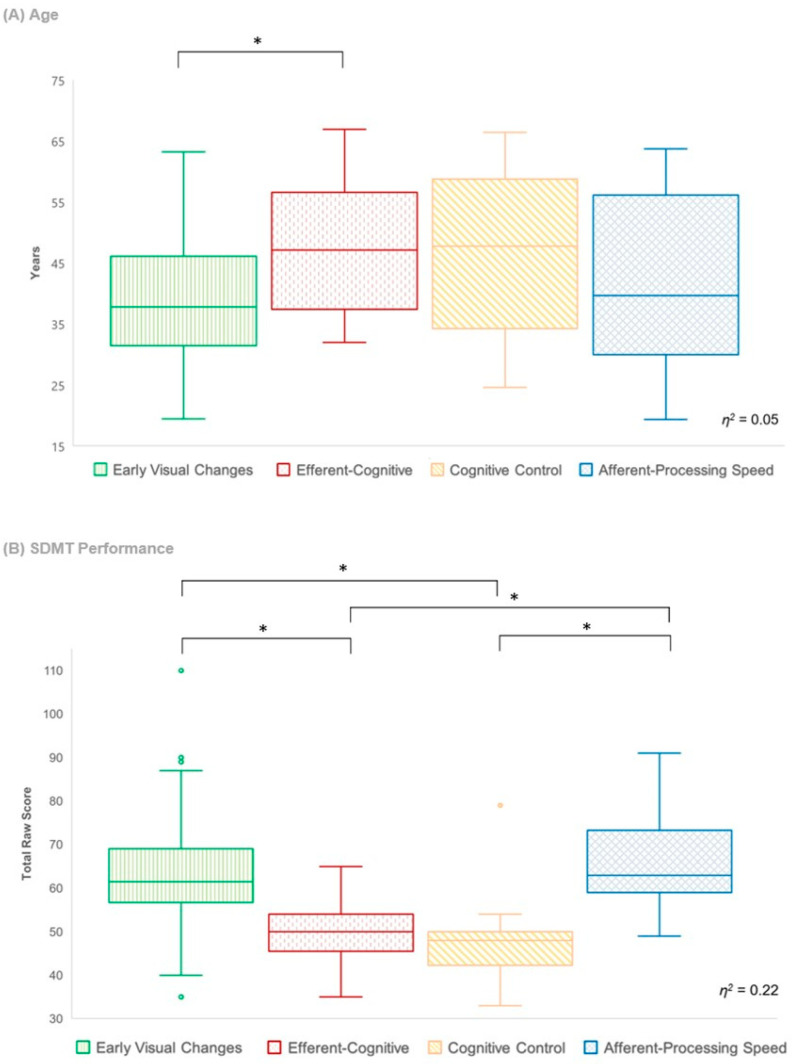
**Inter-phenotype differences in clinical characteristics.** (**A**) Difference in age between phenotypes. (**B**) Difference in performance on the Symbol Digit Modalities Test (SDMT) between phenotypes. * *p* < .05.

The efferent-cognitive phenotype was present in significantly older patients than was the early visual changes phenotype (*χ*^2^ (3) = 9.3, *p* = .03, *η*^2^ = 0.05); however, there were no other significant differences in age between profiles. There were also no significant differences between the four phenotypes with regard to sex, estimated premorbid IQ, self-reported depression, visual acuity, symptom duration and EDSS score, indicating that these factors did not contribute to the differences seen between profiles on visual processing measures. A significant difference in SDMT performance was found between the four profiles (see Figure 3), with the efferent-cognitive and cognitive control phenotypes performing significantly poorer than did the afferent-processing speed and early visual changes phenotypes (*χ*^2^ (3) = 33.42, *p* < .001, *η*^2^ = 0.22). There were neither significant differences in SDMT performance between the efferent-cognitive and cognitive control phenotypes, nor a difference in performance between the afferent-processing speed and early visual changes phenotypes.

## 4. Discussion

Employing a novel three-system framework of afferent, cognitive and efferent visual processing, this exploratory study sought to identify unique visuo-cognitive phenotypes in early MS to examine both the independent and collective impact of visual processing deficits on cognition. This approach allowed for a nuanced examination of the relationship between visual processing and cognition in the early stages of disease, extending beyond the scope of conventional neuropsychological evaluation. Four unique visuo-cognitive phenotypes were identified that differed across each system of visual processing, including the early visual changes phenotype (71.4% of total sample), efferent-cognitive phenotype (8.6% of total sample), cognitive control phenotype (8.6% of total sample) and afferent-processing speed phenotype (11.4% of total sample). These phenotypes provide preliminary evidence that distinct deficits across each system of visual processing may differentially relate to cognitive function, which has implications for the assessment of cognition and patient-centred care in early disease. 

### 4.1. Early Visual Changes Phenotype

The ‘Early Visual Changes’ phenotype was characterised by individuals who only subtly deviated from healthy controls across each system of visual processing (i.e., a < 1 standard deviation from the control mean). Notably, this phenotype contained the highest proportion of CIS patients and was present in patients of a lower mean age compared to that of patients within other profiles, which may denote a mild, early disease stage whereby visual processing is only subtly affected. The high prevalence of this phenotype in our sample is likely attributable to the relatively mild disease course and limited disability burden in our cohort of early MS patients, which contrasts previous cognitive phenotype studies that did not recruit CIS patients and also included patients with progressive disease courses [7,9,10].

Moreover, cognitive and visual impairment often appear subtly at the onset of disease, tending to worsen over the disease course [40,41]. As such, this phenotype may represent the earliest onset of visual processing changes that will potentially worsen to clinically evident visual processing deficits over time. Indeed, Clough et al. [42] recently examined the evolution of working memory phenotypes in early MS and found that ocular motor assessment detected worsening cognitive visual processing over a two-year period despite no changes in performance on standard neuropsychology evaluation.

### 4.2. Efferent-Cognitive Phenotype

The ‘Efferent-Cognitive’ phenotype was characterised by mild to severe cognitive visual processing deficits across multiple domains, including basic processing speed (VG latency), cognitive control (EC, AS and MG error) and spatial accuracy (AS FEP). Notably, this phenotype showed significant efferent impairment related to subclinical dysconjugacy, and post-LPA analysis revealed that this group contained the highest proportion of patients with clinically diagnosed INO. The efferent-cognitive phenotype also had the highest proportion of CDMS patients (92%) who were significantly older than patients in the early visual changes phenotype. However, there were no significant inter-phenotype differences in symptom duration, which may indicate that this subgroup of patients has less effective pharmacological disease control at present or an increased susceptibility to disease progression.

This phenotype had significantly poorer performance on the SDMT compared to that of the early visual changes and afferent-processing speed phenotypes. This provides corroborative evidence of cognitive impairment in the efferent-cognitive phenotype, as the SDMT is sensitive to pronounced cognitive dysfunction. The efferent-cognitive phenotype may therefore represent individuals with more widespread and active disease across cortical and subcortical brain regions, contributing to broader cognitive and ocular motor dysfunction.

### 4.3. Cognitive Control Phenotype

The ‘Cognitive Control’ phenotype was primarily characterised by moderate to severe deficits across tasks of attentional and inhibitory control (EC, AS and MG error), indicative of executive-based difficulties. Patients in this subgroup did not have significant processing speed deficits and displayed largely intact afferent and efferent visual processing. This phenotype may therefore represent individuals who are experiencing a more stable accumulation of neurodegenerative burden within cognitive networks and a relative absence of significant pathology within afferent and efferent pathways.

The cognitive control phenotype also had significantly poorer performance on the SDMT than did the early visual changes and afferent-processing speed phenotypes. However, performance was largely comparable with the efferent-cognitive phenotype. This suggests that our novel methodology was able to capture nuanced differences between the efferent-cognitive and cognitive control phenotypes that were not detectible using standard neuropsychology assessment.

### 4.4. Afferent-Processing Speed Phenotype

The ‘Afferent-Processing Speed’ phenotype was primarily characterised by a mild structural afferent deficit and mild to moderate deficits in basic and cognitive processing speed (VG, EC, AS and MG latency). Notably, post-LPA analyses did not show any inter-phenotype differences in visual acuity, with a median acuity score of 6/6 for each phenotype. Thus, deficits in the afferent-processing speed phenotype may indicate that the thinning of the retinal nerve fibre layer, in the absence of significant visual acuity deficits, can impact the efficiency of cognitive visual processing. This aligns with a recent metanalysis demonstrating an association between RNFL thinning and poorer visual processing speed in individuals without significant vision impairment [20], although this was measured using neuropsychological tasks susceptible to sensory–motor confounders. Costa et al. [22] propose that impairment detected using visual-based measures of processing speed likely relate more to disruptions in the visual system than higher-order cognitive impairment in MS. Our findings may therefore suggest that, in some instances, performance on cognitive outcome measures is influenced or predominantly driven by problems with afferent visual processing rather than cognitive difficulties.

The afferent-processing speed phenotype showed comparable performance to the early visual changes phenotype on the SDMT, despite differing in performance across OM measures of latency. A similar discrepancy was previously reported by Clough et al. [17] who observed poorer cognitive processing speed in MS patients compared to that of controls using ocular motor assessment despite no group differences in SDMT performance being observed. This is likely owed to the multifactorial nature of standard neuropsychological tasks, which involve an array of cognitive and sensory–motor processes. While the oral SDMT is widely used as a measure of processing speed in MS, this task implicates several cognitive functions, making it a sensitive yet indiscriminate measure of cognitive impairment [43] that is also susceptible to visual and motor confounders [11,23]. This common limitation of standard neuropsychological evaluation highlights the importance of conducting factor analyses across tasks to uncover underlying cognitive constructs and implementing a technique that also accounts for the influence of sensory–motor function [44]. In our study, we utilised exploratory factor analysis to understand the latent construct underpinning latency across OM tasks and implemented a method to distinguish cognitive processing speed from sensory–motor processing speed. This approach likely facilitated our identification of the afferent-processing speed phenotype, which was not readily distinguishable from the early visual changes phenotype using the SDMT. This further highlights the nuanced perspective our methodology provides to help disentangle visual processing and cognitive deficits in MS.

Moreover, spatial accuracy on the antisaccade task (AS_FEP) was moderately to severely reduced across the afferent-processing speed, cognitive control and efferent-cognitive phenotypes, while spatial accuracy on the memory-guided task (MG_FEP) appeared largely intact. This discrepancy is consistent with the results of the EFA, which showed that AS_FEP and MG_FEP did not load together on any latent factor. The distinction between spatial accuracy variables across the AS and MG task likely arises from differing task demands. While the MG task primarily relies on working memory to perform a saccade to the spatial location of a previously displayed stimulus, the AS task requires an additional step of vector inversion to perform a saccade in the mirror opposite direction to the original stimulus [45]. Vector inversion is thought to rely on the transmission of spatial information across a widespread network, including bilateral posterior parietal cortices and frontal eye fields, with this increased processing demand contributing to reduced spatial accuracy when performing antisaccades compared to prosaccades [46,47,48]. The transmission of spatial information between hemispheres necessarily requires processing across the corpus callosum, which is a region commonly affected in even milder forms of MS and associated with cognitive impairment [49]. Taken together, the observed deficits in AS spatial accuracy likely represent a more generalised indicator of cognitive impairment in our cohort, perhaps owing to greater corpus callosum-related disease burden across these three phenotypes compared to that in the early visual changes phenotype. However, neuroimaging research is needed to determine the underlying neuropathology associated with these visuo-cognitive phenotypes.

### 4.5. Clinical Characteristics of Phenotypes

Our study did not find any significant differences in symptoms of depression between phenotypes, which contrasts the findings of previous cognitive phenotype studies in MS. For instance, De Meo et al. [7] found that their ‘severe-multidomain’ phenotype had significantly higher depressive symptoms than did other phenotypes in their sample, although this group primarily contained participants with later disease stages and higher physical disability than those of patients in our cohort. Additionally, Podda et al. [9] identified mood disorders as a unique contributor to cognitive phenotypes in their study; however, they assessed cognition using a brief cognitive screening test known to be influenced by depressive symptoms [50]. Contrastingly, our study did not demonstrate an association between depression and any visual processing indicator, including OM variables, which is consistent with previous OM research [19]. This may indicate that the comprehensive evaluation of cognition using OM assessment, combined with objective measures of afferent and efferent visual deficits, is a useful approach for detecting phenotypic differences in cognition that are less influenced by mood-related confounders. However, it is important to note that only 6.5% of our overall sample showed moderate to severe levels of depressive symptoms, suggesting a low prevalence of depression in this study.

More broadly, the lack of significant differences in depression, estimated premorbid IQ, sex, visual acuity, symptom duration and disability severity between the four visuo-cognitive phenotypes suggests that these profiles represent distinct and independent patterns of visual processing impairment. From a clinical perspective, this may indicate that the identified phenotypes are underpinned by different neurological mechanisms, which could be of potential value in tailoring personalised treatment strategies for patients presenting with diverse visual processing deficits. Again, future neuroimaging studies would be required to explore the underlying neurological processes and clinical utility of these phenotypes.

### 4.6. Limitations and Future Directions

The methodological limitations of the current study may restrict the generalisability of our findings to other MS populations. Firstly, our sample size was much smaller than that of other studies that utilised a data-driven method to identify phenotypes in MS [7,9]. While simulation studies in other research areas have recommended large sample sizes for latent profile analysis [38], there is evidence that the inclusion of a greater number of high-quality indicators may help to improve classification accuracy in smaller samples [51]. Utilising a comprehensive array of measures across each system of visual processing, our exploratory study offers initial insights into visuo-cognitive phenotypes in early MS that can be further explored within larger cohorts in future research.

While our study adopted a data-driven approach to classifying individuals into specific phenotypes, we used the threshold of 1 SD to provide qualitative descriptors and make clinical inferences about phenotypes generated via the LPA model. Hancock et al. [10] discovered that setting a threshold of 1 SD versus 1.5 SD significantly affected the phenotypic classification of many individuals in their sample, and although our study did not employ standard deviation cut-offs to classify participants, the application of different cut-off scores may have had the potential to influence the interpretation of profiles in our study. Moreover, the small sample size of our control group, which was used to norm patient data, further limited our ability to delineate visual processing impairment in the patient group. This necessitates closer examination in future research that incorporates larger control groups to establish appropriate cut-off values for determining the severity of impairment for each visual processing indicator, which is critical for phenotype interpretation. Due to the exploratory nature of this research, caution is advised when interpreting the clinical significance of these visuo-cognitive phenotypes.

The measurement of visual acuity represented another limitation in this study. While the Snellen chart is universally employed in clinical settings due to its ease of access and affordability, it has various shortcomings that can hinder its use in a research context. This includes a lack of standardisation in the progression of lines on the chart, as well as inconsistent character counts on different lines, resulting in non-uniform intervals between visual acuity scores [52]. Consequently, incorporating this ordinal variable into our LPA model proved challenging and we were ultimately unable to include a functional measure of afferent visual processing when generating phenotypes. Future studies may consider employing alternative measures of visual acuity that offer greater standardisation and precision such as the Early Treatment Diabetic Retinopathy Study (ETDRS) chart or Bailey–Lovie chart. Notably, these LogMAR charts take longer to administer and are less readily available in clinical settings compared to the Snellen chart, highlighting the trade-off between practicality and precision in data collection.

Finally, the cross-sectional nature of this study does not provide information about the stability of visuo-cognitive phenotypes in MS and the trajectory of visual processing deficits over the disease course. Longitudinal research is therefore needed to track the evolution of these phenotypes over time and examine their association with markers of disease progression.

## 5. Conclusions

This exploratory study endeavoured to better understand the relationship between cognitive and visual processing changes in early MS by employing a novel three-system model of afferent, cognitive and efferent visual processing. Through a comprehensive assessment involving neuro-ophthalmic, ocular motor and neuropsychological evaluation, we were able to delineate four unique visuo-cognitive phenotypes with distinct patterns of visual processing impairment that differentially relate to cognitive function. These findings extend beyond conventional neuropsychological evaluation, providing nuanced insights into the complex relationship between cognitive and visual processing deficits in early MS. By adopting a data-driven approach to examine cognitive constructs across tasks and generate phenotypes, this study also contributes to the growing body of research on cognitive phenotyping in MS. Overall, our findings highlight the need to consider the impact of visual processing impairment when assessing cognition in early MS, with these initial insights holding promise for improving early symptom identification and personalised intervention. Further investigation and validation of these phenotypes is warranted in larger samples that incorporate neuroimaging assessment and a longitudinal study design to uncover the potential neurological mechanisms that underpin these phenotypes and their clinical implications over the disease course.

## Figures and Tables

**Table 1:** Measures completed by patient and control participants.

Measure	Description	Variable(s) of Interest
Afferent Visual Processing		
OCT ^a^	Structural measure of afferent visual pathway	Global RNFL thickness (μm)
Snellen Chart ^a^	Functional measure of afferent visual pathway	Visual acuity score
Cognitive Visual Processing		
Visually Guided Task	Basic prosaccade task primarily measuring simple processing speed	Latency
Endogenously Cued Task	Cognitive OM task primarily measuring processing speed and attentional control	Latency of correct trials and error rate
Antisaccade and Memory Guided Tasks	Cognitive OM tasks primarily measuring processing speed, spatial accuracy, inhibitory control and spatial working memory	Latency of correct trials, error rate and final eye position
Efferent Visual Processing		
VDI	Sub-clinical measure of efferent pathway	Dysconjugacy ratio of abducting and adducting eye on a basic prosaccade task
Ophthalmic Assessment ^a^	Clinical measure of efferent pathway	Diagnosis of INO, nystagmus or oscillopsia
Clinical Characteristics		
Symptom Duration ^a^	Subjective measure of symptom duration	Months since first reported symptom
EDSS ^a^	Measure of disability severity	Total score
Oral SDMT	Screening measure of cognitive impairment	Total raw score
BDI	Screening measure of depressive symptoms	Total score
NART	Estimated premorbid intellectual functioning	Standard score

Abbreviations: OCT, optical coherence tomography; RNFL, retinal nerve fibre layer; OM, ocular motor; VDI, versional dysconjugacy index; INO, internuclear ophthalmoplegia; EDSS, expanded disability status scale; SDMT, Symbol Digit Modalities Test; BDI, Beck Depression Inventory; NART, National Adult Reading Test. ^a^ These measures were only completed by patients during their routine neuro-ophthalmology visit (all other measures were completed by both patients and controls during their research visit).

**Table 2:** Demographic and clinical characteristics of control and patient groups.

	HC (*n* = 25)			CIS (*n* = 50)			CDMS (*n* = 90)			Group Differences		
Sex (F:M)	16:9			39:11			77:13			HC- CIS	HC-CDMS	CIS-CDMS
	*n*	Mean (SD)	Range	*n*	Mean (SD)	Range	*n*	Mean (SD)	Range	*p*		
Age	25	34.24 (13.13)	21–65	50	35.67 (10.01)	19–59	90	43.18 (10.81)	19–66	.86	.001	<.001
NART Std. Score	18	118.22 (3.54)	110–124	49	113.24 (6.4)	96–124	87	115.37 (5.32)	100–126	.004	.12	.08
BDI Total Score	25	3.08 (2.25)	0–8	50	7 (6.15)	0–24	88	7.27 (7.56)	0–38	.04	.02	.97
SDMT Total Score	25	65.66 (12.13)	46.5–97	49	63.76 (13.09)	35–110	85	60.64 (12.53)	33–91	.81	.19	.36
	*n*	Median (IQR)	Range	*n*	Median (IQR)	Range	*n*	Median (IQR)	Range	*p*		
Symptom Duration (mo.)	-	-	-	32	5.5 (19.75)	0–216	64	78.5 (123.25)	4–524	-	-	<.001
EDSS Total Score	-	-	-	43	0 (0)	0–4	76	0 (1)	0–6	-	-	.006
Visual Acuity (LogMAR)	-	-	-	46	0 (0)	−0.12–0.6	80	0 (0)	−0.12–0.3	-	-	.71

Abbreviations: HC, healthy controls; CIS, clinically isolated syndrome; CDMS, clinically definite multiple sclerosis; SD, standard deviation; IQR, interquartile range; NART, National Adult Reading Test; BDI, Beck Depression Inventory; SDMT, Symbol Digit Modalities Test; mo., months; EDSS, Expanded Disability Status Scale; LogMAR, log of minimum angle of resolution.

**Table 3:** Descriptives of visual processing indicators between control and patient groups.

Variable	Healthy Control Group			Patient Group (CIS and CDMS)		
	*n*	Median (IQR) ^a^	Range	*n*	Median (IQR) ^a^	Range
RNFL	-	-	-	124	91.5 (19)	48–121
VG_LATENCY	25	170.06 * (35.16)	134.97–219.89	140	187.86 * (31.73)	133.08–271.26
EC_LATENCY	25	244.81 (61.09)	80.36–380.57	138	247.08 (123.44)	44.07–652.67
EC_ERROR	25	4.17 * (4.17)	0–36.17	140	11.81 * (18.72)	0–50
AS_LATENCY	25	282.38 (161.08)	108.31–497.21	139	300.74 (152.25)	121.79–694.44
AS_FEP	17	21.51 (6.26)	11.87–28.51	138	22.79 (13.35)	5.72–66.23
AS_ERROR	25	6.67 * (4.17)	0–33.33	138	12.5 * (20.53)	0–77.1
MG_LATENCY	24	330.04 (143.23)	155.77–659.24	112	323.37 (134.84)	104.09–844.84
MG_FEP	23	13.32 (7.72)	5.67–28.75	112	12.557 (5.34)	5.28–38.15
MG_ERROR	24	10.42 * (8.1)	2.08–36.17	112	16.7 * (20.9)	0–75
VDI_R	19	1.03 (0.08)	0.86–1.12	136	1.03 (0.12)	0.81–1.57
VDI_L	19	1.04 (0.09)	0.88–1.17	134	1.03 (0.14)	0.65–1.62

Abbreviations: IQR, interquartile range; HC, healthy control; CIS, clinically isolated syndrome; CDMS, clinically definite multiple sclerosis; RNFL, retinal nerve fibre layer thickness; VG, visually guided; EC, endogenously cued; AS, antisaccade; MG, memory guided; FEP, final eye position; VDI_R, rightward versional dysconjugacy index; VDI_L, leftward versional dysconjugacy index. ^a^ Median and interquartile range reported due to the non-normal distribution of the overall patient data. * Significant difference between patients and controls (*p* < .05).

**Table 4:** Four-profile model: phenotype z-scores for each visual processing variable.

Qualitative Phenotype Descriptor	Demographic Characteristics				Profile Estimates ^a^	Afferent	Cognitive									Efferent	
	*n*	F:M	CDMS (%)	Age (*m*)			Basic Processing Speed	Cognitive Processing Speed			Cognitive Control			Spatial Accuracy			
						RNFL (μm)	VG_LAT	EC_LAT	AS_LAT	MG_LAT	EC_ERR	AS_ERR	MG_ERR	AS_FEP	MG_FEP	VDI_R	VDI_L
Early Visual Changes	100	82:18	56	38.72	Mean Est.	−0.809 **	0.676 **	−0.097	−0.144	−0.387 **	0.997 **	0.739 **	0.723 **	0.83 **	−0.241 **	−0.234	0.175
					Median	-	0.489	-	−0.183	-	0.513	0.405	0.083	0.152	−0.345	-	-
					S.E.	0.123	0.115	0.138	0.098	0.073	0.189	0.167	0.188	0.265	0.092	0.147	0.111
Efferent-Cognitive	12	10:2	92	47.78	Mean Est.	−0.921 *	1.347 **	0.089	0.290	−0.273	1.362 **	3.681 **	1.953 **	2.941 **	0.496	6.519 **	0.999
					Median	-	-	-	0.031	−0.347	-	-	-	-	0.241	-	-
					S.E.	0.400	0.268	0.305	0.234	0.167	0.331	0.819	0.555	1.031	0.259	0.588	1.004
Cognitive Control	12	10:2	83	46.73	Mean Est.	−0.921 *	0.818*	0.261	0.650	0.681*	2.770 **	7.415 **	3.826 **	3.231 **	−0.116	−0.797	−0.122
					Median	-	-	-	-	0.436	3.43	-	-	-	-	-	-
					S.E.	0.441	0.375	0.470	0.498	0.267	0.810	1.049	0.744	1.056	0.256	0.537	0.405
Afferent- Processing Speed	16	14:2	81	41.48	Mean Est.	−1.085 **	1.440 **	2.609 **	1.156 **	1.116 **	0.670	0.393	0.717	2.286 *	0.239	0.261	−0.096
					Median	-	-	-	-	-	0.046	−0.201	-	0.984	-	-	−0.417
					S.E.	0.357	0.430	0.494	0.289	0.351	0.360	0.309	0.368	1.069	0.206	0.402	0.376

Abbreviations: F:M, number of females and males; CDMS, clinically definite MS; S.E., standard error of mean estimate; RNFL, retinal nerve fibre layer thickness; LAT, latency; ERR, error; FEP, final eye position; VG, visually guided; EC, endogenously cued; AS, antisaccade; MG, memory guided; VDI_R, rightward versional dysconjugacy index; VDI_L, leftward versional dysconjugacy index. ^a^ Bolded values represent significant mean estimates exceeding the one standard deviation threshold (z-scores > 1 denote impairment for ocular motor variables while z-scores < −1 denote impairment for RNFL). Median values are provided in addition to mean estimates for visual processing indicators with non-normal distributions within each phenotype. * Significant mean estimates within each profile (* *p* < .05 and ** *p* < .01).

## Data Availability

Data has not been made available for this study.

## Supplementary Materials

The following supporting information can be downloaded at: https://www.mdpi.com/article/10.3390/jcm13030649/s1, Figure S1: Visualisation of Missingness across Visual Processing Variables; Figure S2: Density Plots of Imputed Variables in the Multiple Imputation Model; Table S1: Summary of Fit Indices for LPA Models with 1–6 Profiles; Table S2. Sensitivity Analysis: Mean Estimates for 3-Profile Model. References [38,39,53,54,55] are cited in the supplementary materials.
