# Supplementary material for: Visuo-Cognitive Phenotypes in Early Multiple Sclerosis: A Multisystem Model of Visual Processing
Source: J Clin Med. 2024 Jan 23;13(3):649. doi: 10.3390/jcm13030649 (PMC10855997; doi:10.3390/jcm13030649)
Supplement: Supplementary file 1 [file jcm-13-00649-s001.zip › jcm-2813105-supplementary.pdf]

Supplementary Material

S1. Ocular Motor Data Processing

S1.1. Saccadic Metrics

A semi-automated program written in Matlab was used to extract specific saccadic metrics based on time and amplitude coordinates for trial onset, saccade onset, saccade offset and final eye position. This allowed for the detection of the primary saccade within each trial, defined as the first saccade with a velocity of 30 degrees per second that exceeds an amplitude of 1.5 degrees. Manual inspection of the primary saccade in each trial was also conducted to screen for any inaccuracies in the detection of saccadic metrics, as well as any errors made on the task. Trials on a given task were removed from analyses if an error was performed (see below), if there was an unstable baseline fixation (i.e., fixation not maintained within 2.5 degrees of the central target during the first 100ms of trial onset) or if saccade determination was affected by a blink occurring less than 200ms prior to trial onset or during trial onset.

S1.2. Error Classification on Cognitive Tasks

On the endogenously cued (EC) task, an error was defined as a saccade made in the direction indicated by the cue within 100ms of target presentation or to the box opposite the target on invalid trials. Both error rate and latency for valid and invalid trials were combined into a singular EC error and EC latency variable for the purposes of this study. On the antisaccade (AS) task, an error was defined as a saccade made towards the green target cross, as opposed to the diametrically opposite position. On the memory guided (MG) task, an error was defined as a saccade made towards the peripheral red cross or within 100ms of its extinction.

S1.3. Versional Dysconjugacy Index Calculation

Amplitude, defined as the size of the primary saccade, was calculated by measuring the distance between the spatial location of the endpoint (EPoffset) and the spatial location of the starting point (EPonset) of the primary saccade (i.e. EPoffset - EPonset). Velocity, defined as the speed of the primary saccade, was calculated as the distance between the spatial location of the endpoint and starting point of the primary saccade divided by the duration of the saccade (i.e. EPoffset-EPonset/time). The VDI was then calculated for rightward saccades by dividing the average peak velocity over amplitude of the right eye by the average peak velocity over amplitude of the left eye for trials where the target appeared on the right-hand side (i.e., when the right eye is the abducting eye) and vice versa for leftward saccades.

S2. Data Preparation

S2.1. Normalisation of Data & Outlier Detection

Inspection of histograms and the Shapiro-Wilk statistic revealed that RNFL thickness was the only normally distributed visual processing variable in the patient group. Various methods were trialled to normalise the data and handle outliers, including box cox transformation, winsorising of extreme values and the Mahalanobis test for detection of multivariate outliers, as is often recommended when running multiple imputation models or latent profile analyses [38, 53]. While each method helped to

normalise visual processing variables to some extent, it also reduced the clinically meaningful heterogeneity within the sample that was vital for latent profile analyses. These methods of normalisation and multivariate outlier detection are likely more appropriate for larger sample sizes where meaningful variation within a sample can be retained following the application of such techniques. Given that extreme values can significantly bias the results of a latent profile analysis, outliers still needed to be addressed. Ultimately, it was deemed most appropriate to inspect boxplots and histograms for outliers and remove extreme cases from the dataset. Removing only extreme values helped to mitigate outliers biasing profile estimates while also capturing the clinically meaningful variability inherent in the patient group. Outliers were removed for EC latency (2), AS error (1), AS FEP (1), MG latency (1), MG error (1) and VDI_L (2).

S2.2. Missing Data

As depicted in Figure S1, 16 individuals were missing data for RNFL, 14 individuals were missing data for visual acuity, one individual was missing data for AS latency, AS error and AS FEP, 27 individuals were missing data for MG latency and MG error, 28 individuals were missing data for MG FEP and 4 individuals were missing data for VDI_R and VDI_L.

A significantly lower mean RNFL thickness was found in individuals with missing data for MG latency ($m = 84$, $sd = 16.2$, $p = .02$), MG error ($m = 84$, $sd = 16.1$, $p = .02$) and MG FEP ($m = 83.5$, $sd = 15.4$, $p = .01$) compared to individuals who did not have missing values for these indicators ($m = 91.7$, $sd = 13.7$; $m = 91.7$, $sd = 13.7$; $m = 91.8$, $sd = 13.8$). These individuals also had a slightly higher mean VDI_R ($m = 1.1$, $sd = 0.2$, $p = .001$) compared to those without missing data ($m = 1$, $sd = 0.1$). Regarding demographic and clinical characteristics, individuals with missing data for MG latency, MG error and MG FEP were more likely to be male ($p = .04$; $p = .04$; $p = .01$). Those missing data for MG latency and MG error were also older ($m = 45$, $sd = 9.7$, $p = .02$; $m = 44.8$, $sd = 9.5$, $p = .02$) than individuals without missing data ($m = 39.4$, $sd = 11.2$; $m = 39.4$, $sd = 11.3$). Finally, individuals with data missing for visual acuity had a higher mean EDSS score ($m = 1.6$, $sd = 1.6$, $p = .01$) than people without missing data ($m = 0.5$, $sd = 1.1$).

The MG task contained the highest proportion of missingness compared to other visual processing measures, with approximately 20% of observations missing. This was primarily due to a temporary change in protocol where the MG task was not administered. Thus, the significant association between MG variable missingness and various observed variables was likely due to the large proportion of individuals who did not complete this OM task rather than the missing data being attributable to older age, male gender or poorer afferent and efferent visual processing. Taking this into consideration, the mechanism of missingness for MG variables was deemed to be missing at random (MAR).

Concerning the higher mean EDSS score in patients with missing data for visual acuity, various mechanisms of missingness were considered, such as whether these participants were less able or willing to engage in the assessment process, including visual acuity measurement. However, since those missing data for visual acuity did not exhibit any differences in visual processing indicators or other clinical characteristics, this mechanism was considered unlikely. To account for significant associations with missing data, all demographic and clinical variables were added to the imputation model (see section S2.3 for further detail).

Figure S1

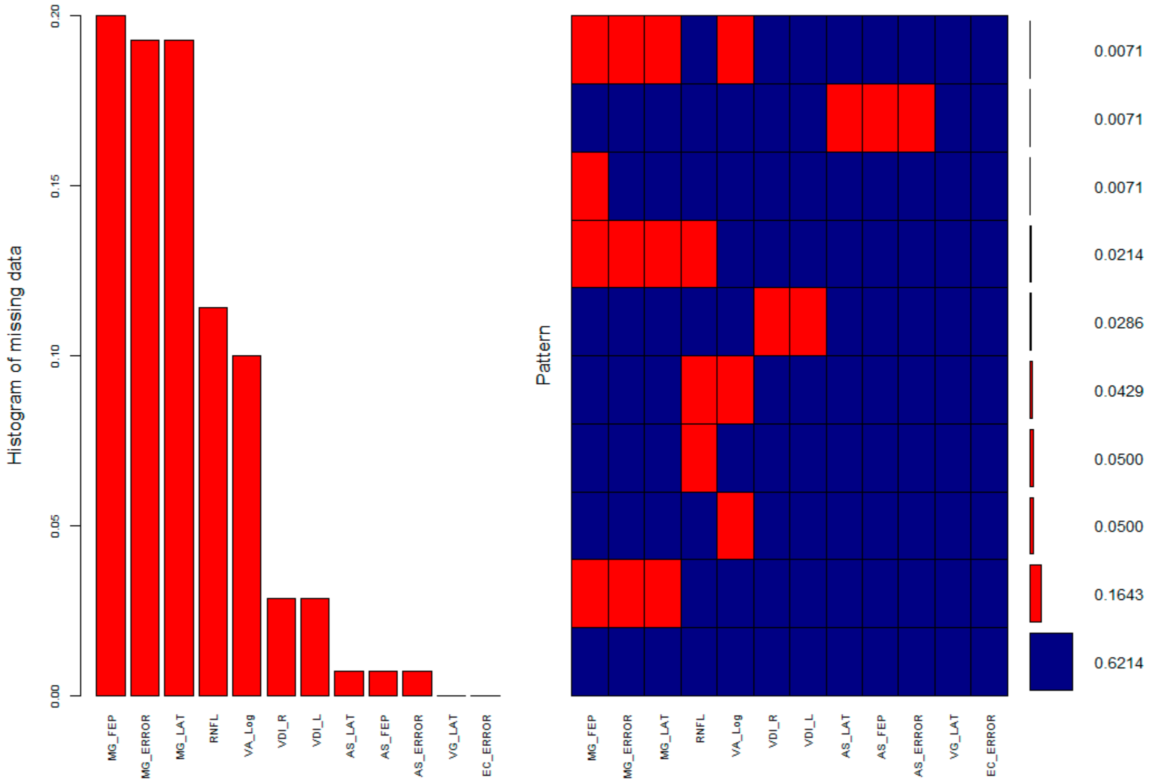

Visualisation of Missingness across Visual Processing Variables. Abbreviations: RNFL, retinal nerve fibre layer thickness; VG, visually guided; EC, endogenously cued; AS, antisaccade; MG, memory guided; FEP, Final Eye Position; VDI_R, rightward versional dysconjugacy index; VDI_L, leftward versional dysconjugacy index, VA_Log, visual acuity on the LogMAR scale.

S2.3. Imputation Model

Multiple imputation was conducted using the aregImpute function from the Hmisc package in R, as this non-parametric method of imputation is robust to non-normal distributions and outliers across both continuous and ordinal scales [53, 54]. Fifty imputed datasets were generated using a model that included all visual processing indicators, as well as the variables of age, sex, symptom duration, NART, BDI, EDSS and SDMT, to improve the estimation of missing values.

Assessment of imputation model fit initially involved the inspection of density plots and strip plots of imputed data. Density plots for each imputed variable showed that the distribution of imputed data generally followed a comparable shape to the distribution of observed data, exhibiting similar peaks and skewness (refer to Figure S2). Strip plots determined that all imputed values were within the range of observed values and there were no outliers detected. Taken together, this suggests that the imputation model was able to generate plausible estimates for missing values. Histograms of imputed values for each missing data point were also inspected for symmetry and imputed values were averaged across the 50 imputed datasets for each missing datapoint. Some imputed distributions were not symmetrical, which may have been influenced by the non-normal distributions of most observed variables in the dataset. Despite the asymmetry of some imputed datapoints, it was deemed appropriate to impute missing data given the other metrics of model fit, in order to reduce the potential bias brought about by missingness in the dataset [55].

Figure S2

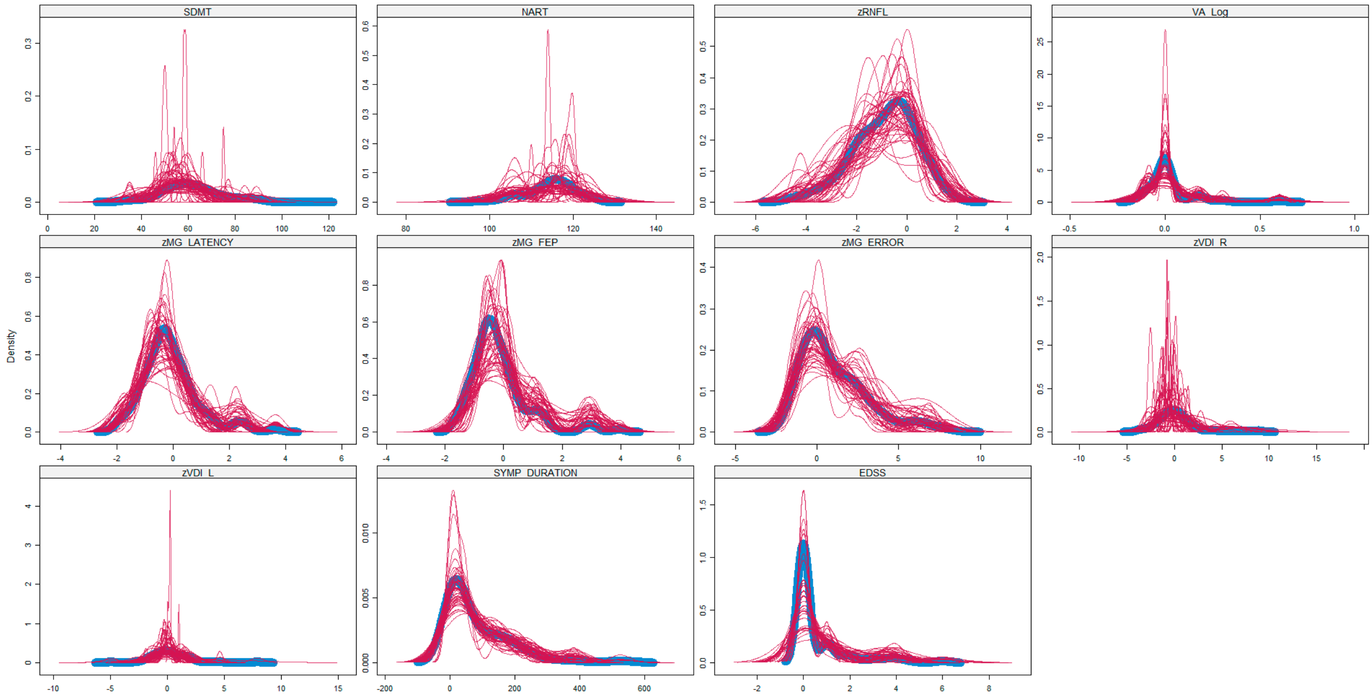

Density Plots of Imputed Variables in the Multiple Imputation Model. The thick blue line represents the density distribution of the observed (non-missing) data, while red lines represent the density distribution of imputed data for each variable. Abbreviations: NART, National Adult Reading Test; BDI, Beck Depression Inventory; SDMT, Symbol Digit Modalities Test, EDSS, Expanded Disability Status Scale; VA_Log, visual acuity on the Log of Minimum Angle of Resolution scale; RNFL, retinal nerve fibre layer thickness; MG, memory guided; FEP, final eye position; VDI_R, rightward versional dysconjugacy index; VDI_L, leftward versional dysconjugacy index.

S3. Exploratory Factor Analysis (EFA)

S3.1. Variable Selection

Initially, VDI_R and VDI_L were included as separate indicators in EFA models, however fit indices were poor. Given that VDI_R and VDI_L were generated using a similar calculation on the same task, this suggested that these variables may not be sufficiently distinct to capture separate dimensions of versional dysconjugacy in the factor analysis. Thus, VDI_R and VDI_L were collapsed into a single averaged indicator for the EFA, which improved fit indices for most factor models. This contrasts the LPA models where VDI_R and VDI_L were able to be included as separate indicators, which allowed for the detection of individuals with unilateral versus bilateral dysconjugacy.

S3.2. Model Selection

The 3-factor model demonstrated better fit indices compared to the 1-factor and 2-factor models. Additionally, each of the three factors had significant factor loadings greater than 0.4 and no variable had significant loadings on more than one factor, indicating that these factors were well-defined and interpretable. While the 4- and 5-factor models had comparably better fit indices to the 3-factor model, some factors did not have any significant indicator loadings, suggesting overfitting of these models. Moreover, the 6-factor model did not converge. Thus, the 3-factor model provided the best fit for the data and most interpretable solution for the underlying structure of ocular-motor variables.

S4. Latent Profile Analysis (LPA)

S4.1. Model Selection

LPA models with 1-5 profiles ran without computational issues and had acceptable entropy (i.e. >.8), as outlined in Table S1. However, the 6-profile model failed to replicate the best log-likelihood value, even after increasing the number of random starts, suggesting potential inaccuracies in profile estimation due to local maxima. Consequently, the 6-profile model was excluded from model selection. The Bayesian information criterion (BIC) indicated that the 4-profile model provided the best fit to the data. While the Bootstrap Likelihood Ratio Test (BLRT) indicated a superior fit of the 5-profile model, the smallest profile in this model comprised only 2% of total participants, which raised uncertainty about whether this represents a unique subgroup that can be generalised to wider populations [39]. Moreover, the 4-profile model had stronger theoretical support than the 5-profile model. The 4-profile model was chosen as the best fit for the data as it had the lowest BIC, a significant BLRT compared to the 3-profile model, greater theoretical alignment than the 5-profile model and all profiles comprising more than 5% of the total sample.

Table S1. Summary of Fit Indices for LPA Models with 1-6 Profiles.

Model	BIC	Loglikelihood	BLRT (*p*)	Entropy	Smallest Profile %
1-Profile	6280.92	-3081.16	-	-	-
2-Profile	6212.26	-3014.71	<.001	.93	16.4%
3-Profile	6206.69	-2979.802	<.001	.96	8.6%
4-Profile	6205.23	-2946.96	<.001	.94	8.6%
5-Profile	6216.67	-2920.55	<.001	.96	2%
[a]6-Profile	6238.24	-2899.216	0.04	.9	2%

Abbreviations: BIC, Bayesian information criterion; BLRT, bootstrap likelihood ratio test. [a] The best loglikelihood value could not be replicated for this model.

S4.2. Sensitivity Analysis

Given the asymmetrical distribution of imputed values for some missing datapoints, a sensitivity analysis was conducted with pairwise missingness using maximum likelihood methods. Similar to the imputed models, LPA models with 1 to 6 profiles were sequentially run and model fit indices were examined to determine which model provided the best fit to the data. A 3-profile model was most appropriate for the non-imputed data, with profile estimates summarised in Table S2. When examining both mean and median estimates, the first class was comparable to the early visual changes phenotype, which only subtly deviated from healthy controls across afferent, cognitive and efferent visual processing measures. The second profile largely resembled the efferent-cognitive profile, containing deficits across both cognitive and efferent visual processing. The final profile appeared to have collapsed the cognitive control and afferent-processing speed phenotypes into a singular profile, with deficits across both latency and error on OM tasks, as well as a mild but significant structural afferent deficit. Considering the small number of participants classified into profiles 2 and 3 of the sensitivity analysis, coupled with the overall comparability of results with the imputed model, the imputed 4-profile model was retained to avoid possible bias brought about by missingness in the data.

Table S2. Sensitivity Analysis: Mean Estimates for 3-Profile Model

Profile	*n*	Profile Estimates [a]	Afferent	Cognitive									Efferent	
				Basic Processing Speed	Cognitive Processing Speed			Cognitive Control			Spatial Accuracy			
			RNFL (µm)	VG_LAT	EC_LAT	AS_LAT	MG_LAT	EC_ERR	AS_ERR	MG_ERR	AS_FEP	MG_FEP	VDI_R	VDI_L
1	121	Mean Est.	-0.835**	0.732**	0.182	0.007	-0.279**	1.003**	0.888**	0.749**	1.069**	-0.207*	0.057	0.022
		Median	-	0.489	0.028	-0.121	-	0.513	0.439	0.041	0.175	-0.374	-0.034	0.037
		S.E.	0.119	0.104	0.138	0.092	0.079	0.169	0.203	0.192	0.270	0.091	0.174	0.112
2	8	Mean Est.	-1.025	1.926**	0.295	0.317	1.044	0.962**	3.450**	1.962	3.314**	0.485	6.858**	3.837**
		Median	- 0.44	1.99	0.144	0.031	1.29	0.91	2.7	0.041	3.65	- 0.596	7.06	3.87
		S.E.	0.644	0.308	0.392	0.329	0.577	0.314	1.264	1.577	0.927	0.972	0.829	0.821
3	11	Mean Est.	-1.020*	1.146**	1.068	1.040	1.394**	2.675**	6.906**	4.137**	3.101**	-0.042	-0.922	-0.304
		Median	-1.216	-	-	-	0.86	-	-	-	-	-0.03	-	-
		S.E.	0.484	0.411	0.674	0.595	0.470	0.783	1.233	0.879	1.039	0.333	0.484	0.365

Abbreviations: S.E., standard error of mean estimate, RNFL, retinal nerve fibre layer thickness; VG, visually guided; EC, endogenously cued; AS, antisaccade; MG, memory guided; FEP, final eye position; VDI_R, rightward versional dysconjugacy index; VDI_L, leftward versional dysconjugacy index. [a]Median values are provided in addition to mean estimates for indicators with non-normal distributions or n<10 within each phenotype. *Significant mean estimates within each profile (**p*<.05, ***p*<.01).
